# Association of early aspirin use with 90-day mortality in patients with sepsis: an PSM analysis of the MIMIC-IV database

**DOI:** 10.3389/fphar.2024.1475414

**Published:** 2025-01-09

**Authors:** Chunsheng Huang, Qiaoling Tong, Wenyuan Zhang, Zhihao Pan

**Affiliations:** ^1^ Department of Anesthesiology, Ningbo Medical Center Lihuili Hospital, Medical School of Ningbo University, Ningbo, China; ^2^ Department of Otolaryngology, Ningbo No.2 Hospital, Ningbo, China; ^3^ The Children’s Hospital, Zhejiang University School of Medicine, National Clinical Research Center for Child Health, Hangzhou, China

**Keywords:** aspirin, sepsis, intensive care unit, 90-day mortality, propensity score matching

## Abstract

**Objective:**

In addition to its antiplatelet and anti-inflammatory properties, aspirin inhibits bacterial proliferation directly. The potential benefits of aspirin may enhance the prognosis for sepsis patients. However, little is known about the effects of early aspirin administration. This study aimed to examine the correlation between the administration of aspirin at an early stage and the 90-day mortality rate among sepsis patients.

**Methods:**

In order to distinguish between septic patients who received early aspirin treatment and those who did not, queries were conducted on the Medical Information Mart for Intensive Care IV (MIMIC-IV) database. The principal metric utilized was 90-day mortality. We determined the association between early aspirin use and 90-day mortality using multivariate Cox regression, and propensity score matching (PSM) was utilized to validate our findings. The analyses of the subgroups have been completed.

**Results:**

Our analysis comprised 28,425 septic patients, of whom 7,568 (26.6%) received aspirin within 24 h of intensive care unit (ICU) admission. The aspirin users group had a lower 90-day mortality than the aspirin nonusers group [1,624 (21.8%) vs. 2,035 (27.3%), *P* < 0.001]. The logistic regression showed that early aspirin use was associated with a lower 90-day mortality (OR, 0.74, 95% CI, 0.69–0.80, *P* < 0.001). K-M curve analysis showed that the 90-day mortality of the aspirin users group was significantly lower than that of the aspirin nonusers group (*P* < 0.001). Subgroup analysis revealed comparable relationships between early aspirin use and 90-day mortality among individuals.

**Conclusion:**

In conclusion, early aspirin use was associated with decreased in-hospital and 90-day mortality in septic patients, emphasizing the significance of early aspirin use administration in the ICU.

## Introduction

Sepsis is the dysfunction of an organ resulting from an infection induced by the pathogen’s disease (Evans et al., 2021). Every year, approximately 48 million patients worldwide are diagnosed with sepsis, which is responsible for as many as 11 million deaths; sepsis has emerged as the primary cause of death among critically ill patients (Rudd et al., 2020). In the past two decades, sepsis mortality has decreased progressively due to the prompt administration of antibiotics, fluids for resuscitation, and multiple organ support therapies. Still substantial mortality exists, and there is scope for enhancement (Seymour et al., 2019; Kempker and Martin, 2020; Fleischmann-Struzek et al., 2020). Survivors of sepsis who are readmitted to the intensive care unit experience substantial functional impairment and a decline in their quality of life (Cheung et al., 2006). Both conditions impose substantial personal and financial burdens on families and society due to the necessity for personal care expenses and employment loss (Rudd et al., 2020; GBD, 2016 Causes of Death Collaborators, 2017).

Despite the progress made in the field of critical care, the fundamental aspects of sepsis management continue to revolve around organ support and infection control (Dellinger et al., 2012). The investigation of the correlation between thrombus, inflammation, and platelets, as well as the potential therapeutic advantages of antiplatelet medications in sepsis, has emerged as a prominent subject of scholarly inquiry (Toner et al., 2015). Acetylsalicylic acid (ASA), often known as aspirin, is a medication that shows promise in treating sepsis. Aspirin exhibits anti-inflammatory and antiplatelet properties, and has been suggested as a potential antisepsis drug due to its direct inhibition of bacterial growth (Menter and Bresalier, 2023; Van Oosterom et al., 2022; Patrono, 2023). The clinical evidence on the efficacy of aspirin in sepsis is inconclusive. Aspirin has been observed to decrease serum levels of inflammatory markers, sepsis-related multiorgan failure (MOF), and mortality in clinical investigations (Hsu et al., 2022; Boyle et al., 2015). A comprehensive cohort research employing propensity-matched design revealed a significant association between prehospital antiplatelet medication and reduced fatality rates (Chow et al., 2022). One randomized clinical investigation, however, revealed that the utilization of aspirin, in comparison to a placebo, did not result in a reduction in the likelihood of developing acute respiratory distress syndrome (ARDS) (Kor et al., 2016). The study’s findings may be constrained by a restricted sample size and may be influenced by uncontrolled confounding variables, such as pre-existing problems, additional life support interventions, and the timing of aspirin administration.

Propensity score matching (PSM) approaches are employed in order to mitigate the influence of sample selection bias and replicate the randomization process (Ryan, 2021; Narduzzi et al., 2014; Austin and Stuart, 2015). The objective of this study is to ascertain whether the early utilization of aspirin is a standalone indicator of mortality in individuals with sepsis. The current study utilized the PSM to examine the influence of early aspirin administration on the 90-day mortality of patients with sepsis.

## Methods

### Study design

A retrospective cohort analysis was conducted utilizing the Medical Information Mart for Intensive Care IV (MIMIC-IV), a prominent database renowned for its origins in the United States (Johnson et al., 2023). The MIMIC-IV database contains complete and high-quality patient information who were admitted to the intensive care units (ICUs) at Beth Israel Deaconess Medical Center. ZWY, one of the writers, mastered the required training to utilize the database and obtained the certificate (certified number 41767848). Individual patient consent was waived due to the project’s lack of impact on clinical care and the anonymization of all protected health information.

### Selection of participants

**FIGURE 1:**
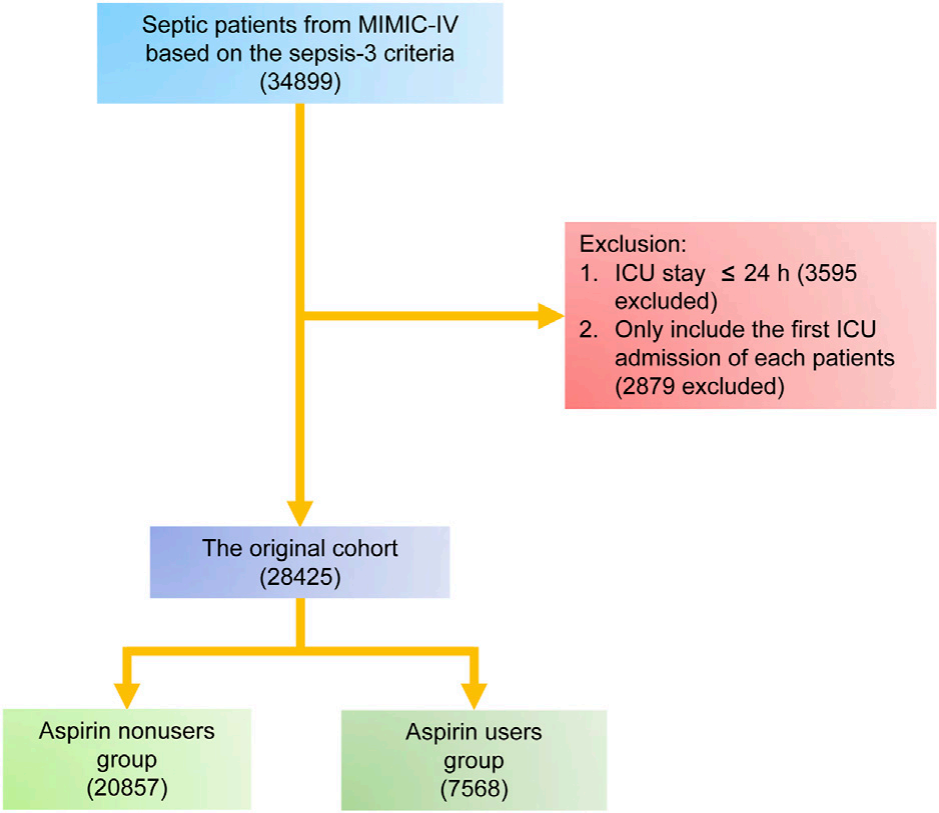
Screening of admissions for inclusion.

The study included patients in the MIMIC-IV who satisfied the criteria for sepsis and were eligible to participate. The sepsis was determined to be present based on the Third International Consensus Definitions for Sepsis and Septic Shock (Sepsis-3) criteria (Singer et al., 2016). In summary, sepsis was identified in persons who had confirmed or suspected infection and saw a sudden increase in their overall Sequential Organ Failure Assessment (SOFA) score of at least two points. Infection was identified by the MIMIC-IV system using the International Classification of Diseases, ninth Edition (ICD-9) code. The study incorporated data from the initial admission to the intensive care unit (ICU) in cases where a patient has been admitted many times. Patients who had an ICU stay of less than 24 h were excluded from the study. Patients who were administered aspirin during a 24 h period following their admission to the intensive care unit (ICU) were categorized into the group of aspirin users, while the remainder patients were categorized into the group of aspirin nonusers. The patient enrollment method of this study is illustrated in Figure 1.

### Variable extraction

Using structured query language (SQL), baseline characteristics within the first 24 h after ICU admission were obtained, including age, gender, weight, race, and insurance. Severity at admission as measured by SOFA score. The use of mechanical ventilation (MV), administration of vasoactive agent, application of extracorporeal membrane oxygenation (ECMO) and continuous renal replacement therapy (CRRT) were recorded. Comorbidities including hypertension, diabetes, myocardial infarction (MI), congestive heart failure (CHF), cerebrovascular disease, chronic obstructive pulmonary disease (COPD), liver disease, renal disease, malignancy, and acquired immune deficiency syndrome (AIDS) were also collected for analysis based on the recorded ICD-9 codes in the MIMIC-IV database. Vital signs included the heart rate (HR), respiratory rate (RR), systolic blood pressure (MBP), Diastolic blood pressure (DBP), temperature and saturation of pulse oxygen (SpO_2_). Laboratory variables including white blood cell (WBC) count, haemoglobin (HBG), platelet (PLT) count, international normalized ratio (INR), sodium, potassium, calcium, anion gap (AG), blood glucose (Glu), creatinine (Cr), and blood urea nitrogen (BUN) were measured during the first 24 h in the ICU. The primary outcome in the present study was 90-day mortality. The secondary outcomes were length of ICU stay, death in hospital and 30-day mortality.

### Statistical analysis

There was no occurrence of data loss for categorical variables. The loss rate of continuous variables was below 10%, resulting in the substitution of mean values for the missing information. The normal distribution of all continuous variables was assessed using the Anderson-Darling test. The medians [interquartile ranges (IQRs)] are used to represent continuous data, whereas total numbers and percentages are used to represent categorical variables. The X^2^ test was employed to compare groups with categorical variables, whereas the Mann-Whitney U test was utilized for continuous variables, as deemed suitable.

The original cohort consisted of the whole number of initial participants. PSM was employed to generate balanced groupings, specifically the matched cohort, in conjunction with the original cohort. A non-parsimonious multivariable logistic regression model was used to calculate the propensity score. The dependent variable was early aspirin use, whereas other baseline parameters were considered as independent factors. The individuals belonging to the group of aspirin users were paired with individuals from the group of aspirin nonusers using the greedy nearest neighbor matching technique, employing a caliper width of 0.2. We implemented PSM changes to validate the accuracy of our findings (Ryan, 2021; Narduzzi et al., 2014; Austin and Stuart, 2015). The effectiveness of PSM was evaluated by calculating standardized mean differences (SMD). SMD plots offer a clear and interpretable method for assessing covariate balance between treated and untreated groups. Since SMD is independent of the unit of measurement, it enables comparisons across variables with different scales, making it especially useful for visual diagnostics (Zhang et al., 2019). A standard deviation of less than 0.1 is seen as a reasonable compromise between the parties (Austin, 2009).

Statistical analysis was conducted using R software (version 4.1.1), with a significance level of *P* < 0.05.

## Results

### Baseline characteristics

**FIGURE 2:**
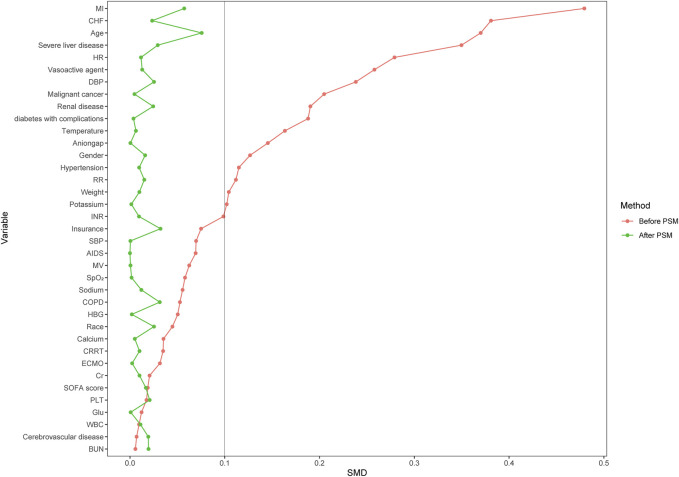
SMD between the aspirin nonusers group and aspirin users group in each cohort.

A total of 28,425 patients were included in this study, with 7,568 individuals in the group of aspirin users and 20,857 patients in the group of aspirin nonusers. The median age of the study patients was 66.42 [54.83–77.92] years old, and 11,687 (56.0%) were males. In total, the 90-day mortality was 27.6%, 3,462 (16.6%) patients died in the hospital and the median length of ICU stay was 3.33 [1.96–6.82] days. After PSM, 7,459 patients in the aspirin users group and 7,459 patients in the aspirin nonusers group were enrolled in the PSM cohort (Table 1). Before matching, the majority of factors between the two groups were not balanced. After matching, the imbalanced covariates were balanced in the matched cohort (Table 2; Figure 2).

**TABLE 1:** Baseline characteristics of subjects in the original and matched cohorts.

Covariates	Before PSM			After PSM		
	Aspirin nonusers	Aspirin users	SMD	Aspirin nonusers	Aspirin users	SMD
N	20,857	7,568		7,459	7,459	
Characteristics						
Age (Years)	66.42 (54.83–77.92)	71.31 (62.49–80.24)	0.370	72.83 (62.72–82.42)	71.27 (62.41–80.23)	0.076
Male (%)	11,687 (56.0%)	4,711 (62.2%)	0.127	4,568 (61.2%)	4,626 (62.0%)	0.016
Weight (Kg)	79.20 (66.00–95.10)	82.60 (69.20–98.20)	0.104	81.00 (67.80–97.40)	82.40 (69.10–98.00)	0.010
Race (%)			0.045			0.025
White	14,865 (71.3%)	5,526 (73.0%)		5,473 (73.4%)	5,417 (72.6%)	
Black	3,183 (15.3%)	1,117 (14.8%)		726 (9.7%)	713 (9.6%)	
Yellow	2,117 (10.2%)	713 (9.4%)		208 (2.8%)	212 (2.8%)	
Other/unkown	3,183 (15.3%)	1,117 (14.8%)		1,052 (14.1%)	1,117 (15.0%)	
Insurance (%)	11,462 (55.0%)	4,440 (58.7%)	0.075	2,967 (39.8%)	3,085 (41.4%)	0.032
SOFA score	3.00 (2.00–4.00)	3.00 (2.00–4.00)	0.019	3.00 (2.00–4.00)	3.00 (2.00–4.00)	0.017
Interventions (1st 24 h) (%)						
MV use	9,604 (46.0%)	3,721 (49.2%)	0.063	3,641 (48.8%)	3,643 (48.8%)	0.001
Vasoactive agent use	10,116 (48.5%)	4,634 (61.2%)	0.258	4,495 (60.3%)	4,542 (60.9%)	0.013
ECMO use	42 (0.2%)	28 (0.4%)	0.032	26 (0.3%)	25 (0.3%)	0.002
CRRT use	1,336 (6.4%)	422 (5.6%)	0.035	395 (5.3%)	412 (5.5%)	0.010
Comorbidities (%)						
Hypertension	8,403 (40.3%)	3,479 (46.0%)	0.115	3,465 (46.5%)	3,429 (46.0%)	0.010
Diabetes	1948 (9.3%)	1,174 (15.5%)	0.118	1,138 (15.3%)	1,148 (15.4%)	0.004
MI	2,670 (12.8%)	2,443 (32.3%)	0.479	2,156 (28.9%)	2,352 (31.5%)	0.057
CHF	5,814 (27.9%)	3,476 (45.9%)	0.381	3,478 (46.6%)	3,391 (45.5%)	0.023
Cerebrovascular disease	3,022 (14.5%)	1,078 (14.2%)	0.007	1,116 (15.0%)	1,065 (14.3%)	0.019
COPD	5,726 (27.5%)	2,258 (29.8%)	0.035	2,338 (31.3%)	2,230 (29.9%)	0.031
Liver disease	2021 (9.7%)	129 (1.7%)	0.350	102 (1.4%)	129 (1.7%)	0.029
Renal disease	4,775 (22.9%)	2,370 (31.3%)	0.190	2,407 (32.3%)	2,322 (31.1%)	0.024
Malignancy	3,181 (15.3%)	654 (8.6%)	0.205	644 (8.6%)	654 (8.8%)	0.005
AIDS	214 (1.0%)	33 (0.4%)	0.069	33 (0.4%)	33 (0.4%)	<0.001
Vital signs (1st 24 h)						
HR (Beats/min)	87.00 (76.13–99.15)	82.51 (74.32–92.09)	0.279	82.31 (73.15–92.96)	82.54 (74.35–92.18)	0.012
RR (Beats/min)	19.14 (16.76–22.20)	18.75 (16.69–21.41)	0.112	18.69 (16.56–21.50)	18.74 (16.69–21.41)	0.015
SBP (mmHg)	113.00 (104.25–124.86)	112.31 (105.19–121.12)	0.070	112.23 (104.17–122.74)	112.39 (105.22–121.25)	<0.001
DBP (mmHg)	60.83 (54.82–68.04)	58.74 (53.40–64.63)	0.238	58.48 (52.83–64.92)	58.78 (53.44–64.68)	0.025
Temperature (°C)	36.87 (36.61–37.23)	36.80 (36.56–37.09)	0.163	36.81 (36.55–37.12)	36.81 (36.56–37.09)	0.006
SpO_2_ (%)	97.25 (95.77–98.59)	97.40 (96.00–98.55)	0.058	97.39 (95.93–98.65)	97.40 (96.00–98.55)	0.002
Laboratory tests (1st 24 h)						
WBC (×10^9^/L)	13.40 (9.40–18.50)	14.10 (10.50–18.60)	0.010	13.60 (9.70–18.60)	14.10 (10.50–18.50)	0.011
HBG (g/dL)	9.60 (8.20–11.20)	9.50 (8.20–10.90)	0.050	9.50 (8.20–10.90)	9.50 (8.20–10.90)	0.002
PLT (×10^9^/L)	164.00 (107.00–233.00)	158.00 (117.00–219.00)	0.017	166.00 (116.00–226.00)	158.00 (118.00–219.00)	0.021
INR	1.2000 (1.1000–1.5000)	1.2000 (1.1000–1.4000)	0.099	1.20 (1.10–1.40)	1.20 (1.10–1.40)	0.010
Sodium (mmol/L)	140.00 (137.00–143.00)	140.00 (137.00–142.00)	0.056	140.00 (137.00–142.00)	140.00 (137.00–142.00)	0.012
Potassium (mmol/L)	4.50 (4.10–5.00)	4.60 (4.20–5.00)	0.102	4.50 (4.10–5.10)	4.60 (4.20–5.00)	0.001
Calcium (mg/dL)	8.50 (8.00–9.00)	8.50 (8.10–9.00)	0.035	8.50 (8.00–9.00)	8.50 (8.10–9.00)	0.005
AG (mmHg)	16.00 (14.00–19.00)	15.00 (13.00–19.00)	0.145	16.00 (13.00–19.00)	15.00 (13.00–19.00)	<0.001
Glu (mg/dL)	131.11 (111.00–161.17)	132.00 (118.14–160.83)	0.012	132.67 (114.00–161.77)	131.92 (118.00–160.82)	0.001
Cr (mg/dL)	1.20 (0.80–1.90)	1.20 (0.90–1.90)	0.021	1.20 (0.90–2.00)	1.20 (0.90–2.00)	0.010
BUN (mg/dL)	24.00 (16.00–40.00)	24.00 (16.00–40.00)	0.006	25.00 (17.00–41.00)	24.00 (16.00–40.00)	0.020

SOFA: sequential organ failure assessment, SAPS II: Simplified Acute Physiology Score II, GCS: glasgow coma scale, MV: mechanical ventilation, ECMO: extracorporeal membrane oxygenation, CRRT: continuous renal replacement therapy, MI: myocardial infarction, CHF: congestive heart failure, COPD: chronic obstructive pulmonary disease, AIDS: acquired immune deficiency syndrome, HR: heart rate, RR: respiratory rate, MBP: mean blood pressure, SpO_2_: saturation of pulse oxygen, WBC: white blood cell count, HCT: haematocrit, HBG: haemoglobin, PLT: platelet counts, INR: international normalized ratio, AG: anion gap, Glu: blood glucose, Cr: creatinine, BUN: blood urea nitrogen.

**TABLE 2:** Outcomes of subjects in the original and matched cohorts.

Covariates	Before PSM			After PSM		
	Aspirin nonusers	Aspirin users	*P*	Aspirin nonusers	Aspirin users	*P*
N	20,857	7,568		7,459	7,459	
Length of ICU stay	3.33 (1.96–6.82)	2.70 (1.49–5.17)	<0.001	3.30 (1.97–6.59)	2.65 (1.48–5.07)	<0.001
Death in hospital	3,462 (16.6%)	903 (11.9%)	<0.001	1,171 (15.7%)	903 (12.1%)	<0.001
30-day mortality	4,185 (20.1%)	1,164 (15.4%)	<0.001	1,453 (19.5%)	1,164 (15.6%)	<0.001
90-day mortality	5,761 (27.6%)	1,624 (21.5%)	<0.001	2035 (27.3%)	1,624 (21.8%)	<0.001

### The secondary outcomes with different cohorts

In the original cohort, the aspirin users group had a lower length of ICU stay [2.70 [1.49–5.17] days vs. 3.33 [1.96–6.82] days, *P* < 0.001] than the aspirin nonusers group. In the matched cohort, the aspirin users group also had a lower length of ICU stay [2.65 [1.48–5.07] days vs. 3.30 [1.97–6.59] days, *P* < 0.001] than the aspirin nonusers group (Table 2). The chi-square test showed that the aspirin users group had a lower in-hospital mortality [903 (11.9%) vs. 3,462 (16.6%), *P* < 0.001] than the aspirin nonusers group in the original cohort. In the matched cohort, the aspirin users group also had a lower in-hospital mortality than the aspirin nonusers group [903 (12.1%) vs. 1,171 (15.7%), *P* < 0.001]. The aspirin users group had a lower 30-day mortality [1,164 (15.4%) vs. 4,185 (20.1%), *P* < 0.001] than the aspirin nonusers group in the original cohort. In the matched cohort, the aspirin users group also had a lower 30-day mortality than the aspirin nonusers group [1,164 (15.6%) vs. 1,453 (19.5%), *P* < 0.001] (Table 2). The Cox regression in the original cohort showed that early aspirin use was associated with a lower 30-day mortality, with an HR of 0.75 (95% CI, 0.70–0.80, *P* < 0.001). This association remained significant in the matched cohort (HR, 0.79, 95% CI, 0.73–0.85, *P* < 0.001). In the original cohort, K-M curve analysis showed that the 30-day mortality of the aspirin users group was significantly lower than that of the aspirin nonusers group (Supplementary Figure S1A, *P* < 0.001). This result remained significant in the matched cohort (Supplementary Figure S1B, *P* < 0.001).

### 90-day mortality with different cohorts

**FIGURE 3:**
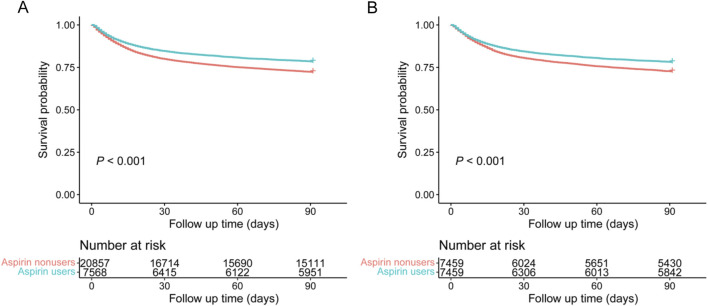
K-M curves were used to compare the 90-day mortality of patients with sepsis between the aspirin nonusers group and aspirin users group in each cohort. **(A)** In the original cohort; **(B)** In the matched cohort.

**FIGURE 4:**
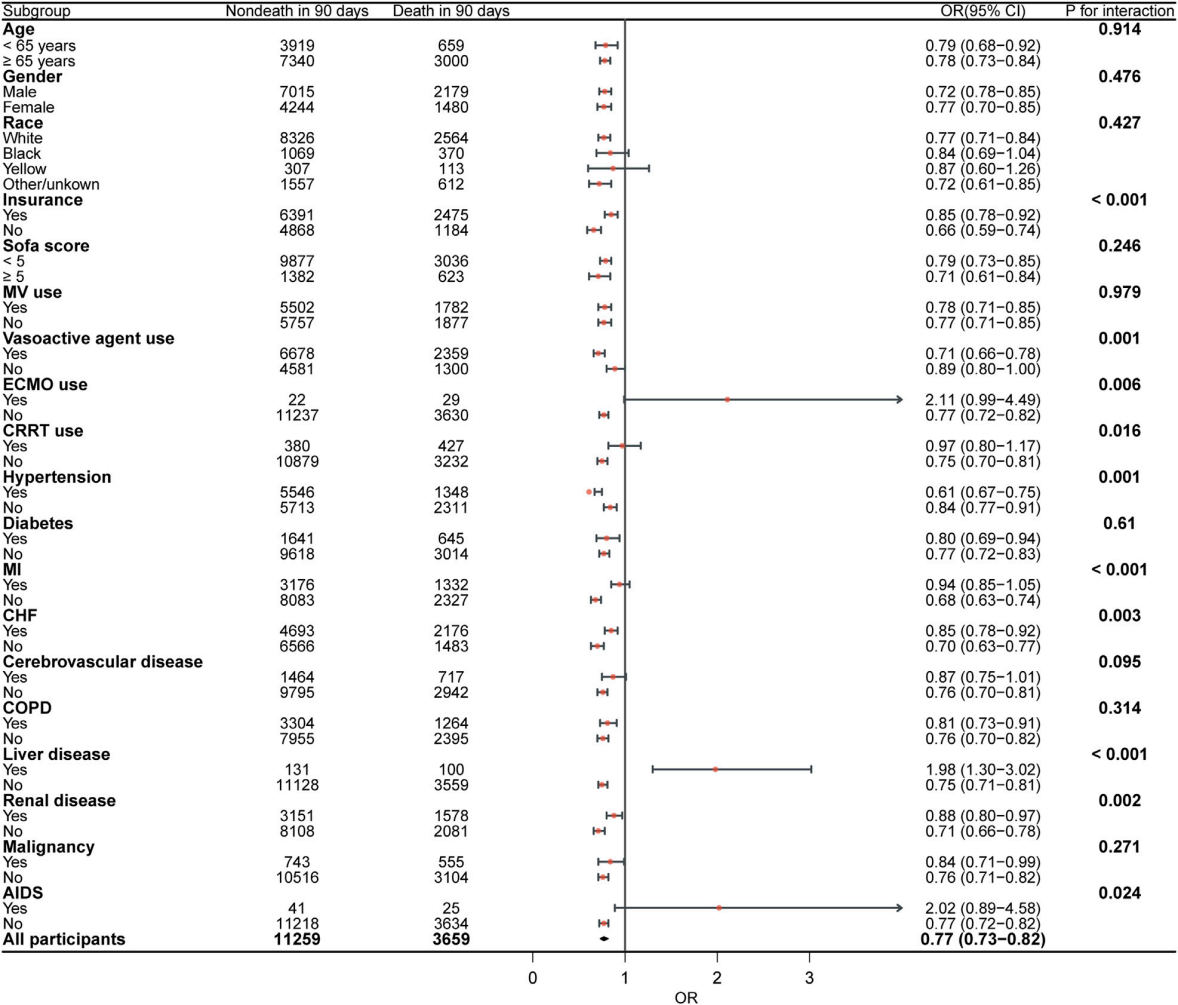
Subgroup analyses of the association between early aspirin use and 90-day mortality.

The chi-square test showed that the aspirin users group had a lower 90-day mortality [1,624 (21.5%) vs. 5,761 (27.6%), *P* < 0.001] than the aspirin nonusers group in the original cohort. In the matched cohort, the aspirin users group had a lower 90-day mortality than the aspirin nonusers group [1,624 (21.8%) vs. 2,035 (27.3%), *P* < 0.001] (Table 2). The Cox regression in the original cohort showed that early aspirin use was associated with a lower 90-day mortality, with an HR of 0.75 (95% CI, 0.71–0.79, *P* < 0.001). This association remained significant in the matched cohort (HR, 0.77, 95% CI, 0.73–0.83, *P* < 0.001). In the original cohort, K-M curve analysis showed that the 90-day mortality of the aspirin users group was significantly lower than that of the aspirin nonusers group (Figure 3A, *P* < 0.001). This result remained significant in the matched cohort (Figure 3B, *P* < 0.001). Subgroup analysis revealed comparable relationships between early aspirin use and 90-day mortality among individuals (Figure 4).

## Discussion

The main aim of this study was to assess the potential association between early treatment of aspirin and enhanced survival outcomes in individuals with sepsis. The justification for investigating aspirin as a novel therapeutic intervention arises from its extensively documented anti-inflammatory and antiplatelet characteristics. In the present study, it was observed that those who were administered aspirin during the initial phases of sepsis demonstrated a notably reduced mortality rate during a 90-day period in comparison to those who did not get aspirin. This observation necessitates additional examination of the potential of aspirin in alleviating the systemic ramifications of sepsis. The results of our study provide valuable insights that contribute to the continuing scholarly conversation on the management of sepsis.

Previous research examining the impact of aspirin on the vulnerability to sepsis and its resulting effects has yielded diverse findings. Using a nested cohort study, Al Harbi, et al. found that aspirin use in ICU patients was associated with increased rates of severe sepsis and length of stay (Al Harbi et al., 2016). The findings of a population-based cohort analysis indicate that there is no significant association between baseline aspirin use and long-term incidence of sepsis (Hsu et al., 2018). In a retrospective population-based cohort analysis, it was observed that the administration of aspirin before to hospital admission is linked to a decreased mortality rate within a 90-day period among patients diagnosed with sepsis (Hsu et al., 2022). These findings align with the outcomes of our research. Our data differed from previous research in that they were not restricted to ICU admission and encompassed data on various baseline participant characteristics. After accounting for demographics and chronic medical disorders, we saw that the strongest reduction in the connection between early aspirin usage and 90-day mortality occurred. This indicates that aspirin use could potentially serve as an indicator of the burden of other medical conditions.

The correlation established between the early initiation of aspirin usage and a decrease in mortality rates presents thought-provoking inquiries on the fundamental mechanisms involved. However, the precise role of aspirin in sepsis remains uncertain, particularly with respect to the dosage and the reasons for its prescription. The vast majority of sepsis patients in this study received low-dose aspirin (e.g., 75 mg per day), which is commonly used for secondary prevention post-myocardial infarction (MI), primarily acting by inhibiting platelet cyclooxygenase (COX-1), which reduces platelet aggregation. This mechanism may offer a plausible explanation for its potential benefit in sepsis, given the recognized role of platelets in the pathogenesis of sepsis-related thrombosis and microvascular dysfunction (Cox, 2023; Yang et al., 2023). Moreover, while anti-platelet agents are not a cure for sepsis, they have shown promise in stabilizing patients by preventing progression to disseminated intravascular coagulation (DIC). This can provide clinicians with valuable time to address the underlying infection, particularly in cases where sepsis is culture-negative, a scenario in which antibiotics may be ineffective in the absence of an identified pathogen. Sepsis is known to induce endothelial dysfunction, leading to vascular leakage and contributing to organ failure (Joffre et al., 2020; Zhang et al., 2023). In this context, the potential protective effects of aspirin on the endothelium have been proposed (Dzeshka et al., 2016; Liu et al., 2023). By modulating the balance between vasoconstrictive and vasodilatory chemicals, aspirin may help preserve endothelial integrity, potentially counteracting the vascular damage seen in sepsis. Furthermore, The immunomodulatory effects of aspirin have been demonstrated, encompassing the modification of immune cell function (Schieffer and Drexler, 2003). This has the potential to impact the equilibrium between pro-inflammatory and anti-inflammatory reactions in sepsis, thereby promoting a more regulated and harmonious immune response.

Despite the importance of our findings, several limitations should be acknowledged. First, the retrospective nature of the study design introduces potential selection bias, although we employed the PSM approach to mitigate this. Additionally, we lacked information on whether patients were on aspirin prior to admission and the specific reasons for its use, which limits our understanding of its mechanisms in sepsis outcomes. Future research should focus on prospectively documenting patients’ medication history, including aspirin use and the clinical indications for its prescription, to better assess its impact on disease progression and treatment outcomes. Furthermore, since our study is based on an observational database, the findings should be interpreted as preliminary. Therefore, future prospective studies, particularly randomized controlled trials comparing aspirin with a placebo in sepsis patients, are necessary to more conclusively determine the causal relationship between aspirin use and mortality.

In conclusion, early aspirin use was associated with decreased 90-day mortality in septic patients, emphasizing the significance of early aspirin use strategy in the ICU.

## Data Availability

Publicly available datasets were analyzed in this study. This data can be found here: https://www.physionet.org/content/mimiciv/3.0/.
