# Supplementary material for: Association of early aspirin use with 90-day mortality in patients with sepsis: an PSM analysis of the MIMIC-IV database
Source: Front Pharmacol. 2025 Jan 9;15:1475414. doi: 10.3389/fphar.2024.1475414 (PMC11754289; doi:10.3389/fphar.2024.1475414)

Supplementary Figure S1: K-M curves were used to compare the 30-day mortality of patients with sepsis between the aspirin nonusers group and aspirin users group in each cohort. (A) In the original cohort; (B) In the matched cohort.


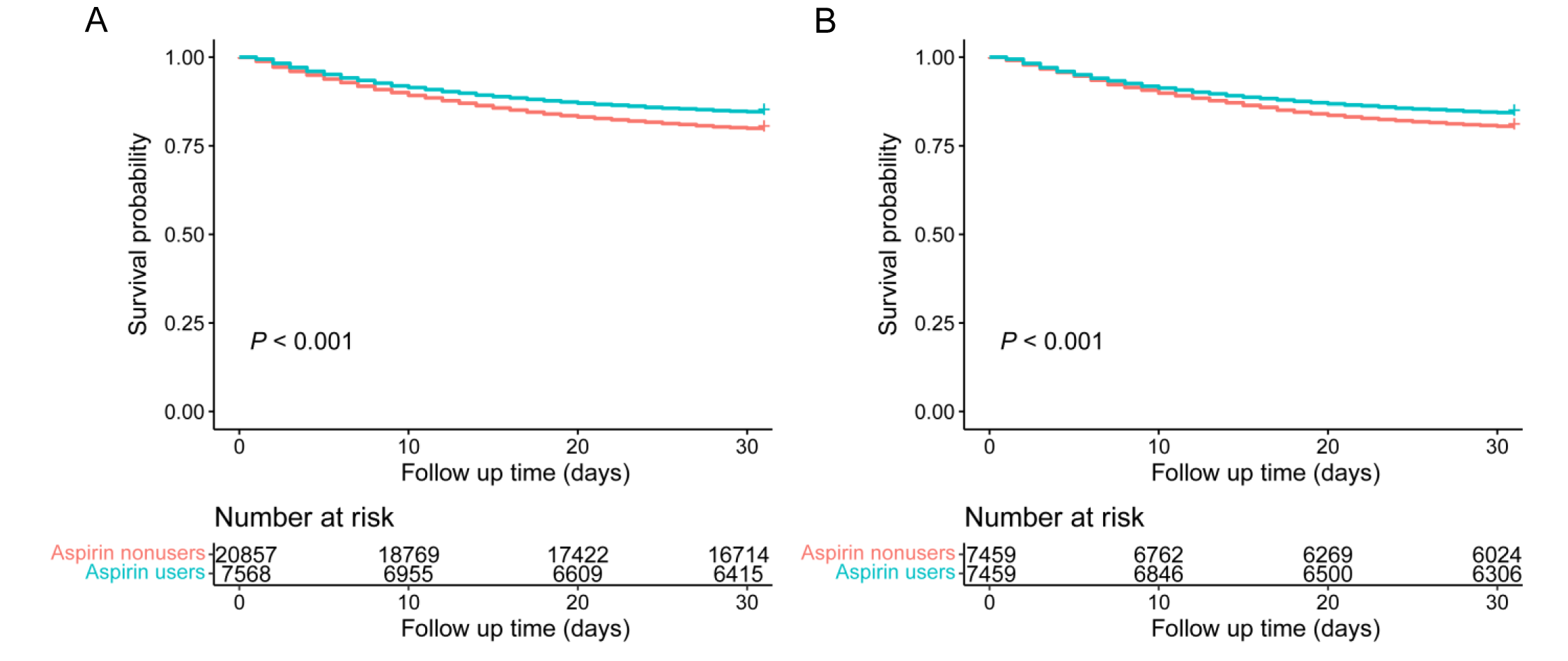

Supplement: Supplementary file 1 [file DataSheet1.docx]
